# The contribution of resident physicians to hospital productivity

**DOI:** 10.1007/s10198-021-01368-z

**Published:** 2021-08-21

**Authors:** Maria J. Perez-Villadóniga, Ana Rodriguez-Alvarez, David Roibas

**Affiliations:** 1 Economics Department, University of Oviedo, Oviedo, Spain; 2 Oviedo Efficiency Group, Oviedo, Spain

**Keywords:** Medical residents, Hospital productivity, Distance functions, Non-linear stochastic frontier models, I18, J24

## Abstract

Resident physicians play a double role in hospital activity. They participate in medical practices and thus, on the one hand, they should be considered as an input. Also, they are medical staff in training and, on the other hand, must be considered as an output. The net effect on hospital activities should therefore be empirically determined. Additionally, when considering their role as active physicians, a natural hypothesis is that resident physicians are not more productive than senior ones. This is a property that standard logarithmic production functions (including Cobb–Douglas and Translog functional forms) cannot verify for the whole technology set. Our main contribution is the development of a Translog modification, which implies the definition of the input “doctors” as a weighted sum of senior and resident physicians, where the weights are estimated from the empirical application. This modification of the standard Translog is able, under suitable parameter restrictions, to verify our main hypothesis across the whole technology set while determining if the net effect of resident physicians in hospitals’ production should be associated to an output or to an input. We estimate the resulting output distance function frontier with a sample of Spanish hospitals. Our findings show that the overall contribution of resident physicians to hospitals’ production allows considering them as an input in most cases. In particular, their average productivity is around 37% of that corresponding to senior physicians.

## Introduction

In many countries, before becoming a physician, medical students must go through a long training process after completing their medical studies. Residency training generally takes place at a teaching hospital, where residents practice medicine under the supervision and instruction of fully licensed physicians. Teaching hospitals provide prospective future doctors with necessary education, which is a public good [1], insofar as well-trained physicians benefit society in general. At the same time, during their training process, residents also contribute to the provision of healthcare services. Given the particular nature of training programs in teaching hospitals, there is an increasing concern regarding the precise effect of medical residency on these hospitals’ performance.

In this sense, residents’ productivity has raised interest among economists. Residents have been considered as both inputs and outputs in hospitals’ production models, as [2] point out. Therefore, in the literature there is not consensus as to the role of residents in hospital production. Several authors (for example [3–5]) model medical residents as outputs, assuming that training introduces more complexity to the activity carried out by senior physicians given that they face the added challenge of training student doctors. However, residents have also been considered as inputs in the literature, given their active participation in clinical practice [6, 7] or [8].

In most cases, the results indicate that teaching activities may reduce other inputs' productivity and increase hospital costs [9, 10]. However, although university hospitals have higher costs, the physician-substitution effect should be also considered [11]. Still, some studies find that residents increase the productivity of attending physicians. For example, [12] examine the impact of learners on emergency medicine attending physicians’ productivity. They find that, when attending physicians are paired with a resident, the number of patients seen per hour increases significantly relative to the situation where the attending physician is alone. This result is somewhat supported by [13], who show that the implementation of an emergency medicine residency program had a positive effect on the clinical efficiency of attending physicians.

More recently, a set of studies exists that use frontier models to capture hospital technology (e.g. [14]). In this way, it is also possible to analyse the role of residents in the provision of patient care. For example, [15] use a DEA approach to compare the frontiers of teaching and non-teaching hospitals, treating residents as inputs. According to their results, only roughly 10% of the teaching hospitals perform better than the non-teaching hospitals. In several studies, [1, 16] use a similar methodology to assess technical inefficiency with a sample of teaching hospitals. They find an average inefficiency score of 0.80, 20% of which could be explained by medical resident congestion. Moreover, congestion appears to be positively correlated to hospital teaching intensity. Analysing the determinants of inefficiency, [17, 18] find that teaching activities increase technical inefficiency. In contrast, [19] do not find a significant relationship between efficiency and teaching status.

In a more recent study, [2] follow a data-driven parametric approach based on the directional technology distance function to determine whether residents are inputs or outputs in the provision of healthcare. Using the American Hospital Association panel data from 1994 to 2010, they show that residents are, on average, inputs in all rural and in public non-teaching hospitals, but outputs in urban area teaching not-for-profit hospitals.

In sum, this debate is still open, as difficulties exist in identifying whether medical residents should be considered as inputs or outputs. The aim of this paper is to shed more light about the role of residents in hospital production. Concretely, we present a model where medical residents are considered simultaneously both as an input and an output and assess their net effect on hospital production. Furthermore, we assume the hypothesis that if the net effect of resident physicians is to be an input, then their contribution to hospital services production is not larger than that of senior physicians.^1^ More particularly, our contribution is the development of a logarithmic model in which, under some testable parameter restrictions, the estimated technology will verify the former hypothesis regardless of the input endowment and the output vector.

Standard logarithmic models (Cobb–Douglas, Translog) imply, as it is shown below, that if residents and senior physicians are considered different inputs then, the marginal rate of substitution between them will be larger than one in some zones of the technology space and lower than one in others. This will imply that resident physicians should be estimated as more productive than the senior ones for some zones of the technology space. This characteristic of the logarithmic models may potentially bias the empirical analysis if our hypothesis is maintained by the data generation process but, as a result of the estimation procedure, an important set of the observations is found to show higher productivity for residents than for senior physicians. Therefore, a model able to verify our main hypothesis for the whole technology set may improve the fit of the empirical model to the true data generation process. Then, the model determines the conditions under which resident physicians’ contribution to hospitals’ production of health services is positive and should be considered as an input (but no more productive than a senior physician in any case) or, on the contrary, they reduce the productivity of senior physicians involved in the teaching of learners in such a way that their contribution to hospitals’ productivity is negative and, therefore, should be considered as an output.

It is worth noting that assessing the dominant effect of resident physicians in health production services could be important to design the optimal contract for resident physicians, including their remuneration. If their effect as educational output dominates their contribution as input, their remuneration can be considered as a grant. On the other hand, if the dominant effect is their contribution as input, residents’ marginal productivity should be taken into account when determining their salary.

We present an empirical application of this model evaluating the impact of resident physicians on Spanish teaching hospitals’ productivity. To do this, we use a Spanish hospital panel data sample for the period 1997 to 2009.

## Methodology

The net effect of resident physicians on hospital production activities depends on whether their contribution as active physicians overcompensate their consumption of inputs in training activities (mainly senior physicians’ time). Our hypothesis is that even if the net effect of residents’ training on hospital production is positive, so that they can be globally considered as an input, they will not be more productive than a senior physician. To incorporate this hypothesis into the empirical analysis, we use a logarithmic function (in particular a translog functional form), and follow a method similar to that used by [20] for identifying gender differences in productivity. To deal with differences in productivity between attending and resident physicians, we define medical labour input as follows:

1 $${x}_{1}=\left({x}_{1}^{S}+\gamma { \left(z\right) x}_{1}^{R}\right),$$

where ${x}_{1}^{S}$ is the number of senior physicians, ${x}_{1}^{R}$ is the number resident physicians, $\gamma \left(z\right)$ is a function that captures the resident physicians’ contribution to hospital services production relative to that of attending physicians, and *z* is a vector of variables that could influence this ratio of productivities. Then, if our maintained assumption regarding the true data generation process whereby resident physicians are not more productive than senior physicians, the value of *γ(z)* is expected to be always equal of lower than one. Hence, a value of *γ(z)* equal to 1 implies that resident physicians are just as productive as senior physicians. If *γ(z)* ranges between 0 and 1, then resident physicians positively contribute to hospital production services and globally should be considered as an input, but their productivity is lower than that of senior physicians. Finally, a value of *γ(z)* below 0 indicates that the net contribution of resident physicians to hospital production of medical services is negative because they consume part of the working time of senior physicians, and therefore should be considered as an output rather than an input.

This characterization of the medical labour input can also be seen in terms of labour augmenting technological change models, where technological progress increases the amount of *effective labour*. In this case, the equivalent of technological progress would be the use of resident physicians, who may contribute to augment or reduce the productivity of senior doctors [21].^2^

In particular, we assume that the differences in productivity between senior and resident physicians may depend on the complexity of the case mix treated in the hospital. This way, we expect that the larger the complexity of the case mix, the lower will be the resident physicians’ productivity relative to that of senior physicians. That is, more simple cases may be treated by a resident physician in the same manner as a senior one. However, complex cases would require a senior physician and the participation of resident physicians may be limited to the learning process itself, demanding working time from senior physicians. Therefore, the function *γ(z)* is defined as:

2 $$ \gamma \left( z \right) = \;\left( {\gamma \; + \;\gamma_{rICU} \;rICU} \right), $$

where $\gamma ;{}_{rICU}$ are parameters to be estimated; $rICU$ is the proportion of intensive care unit (ICU) discharges over the total discharges (excluding ICU discharges) weighted by weighted care units (WCU)^3^ and could be understood as a proxy for the case-mix complexity.^4^

Then, ${\gamma}_{rICU}$ is expected to be negative, reflecting a relatively lower productivity of resident physicians when the case mix becomes more and more complex. Given that our hypothesis is that $\gamma \left(z\right)\le 1$, then a value of $\le 1$ guarantees that Eq. (2) takes a value lower than or equal to one regardless of the value of *rICU*.

Given the multiple output nature of hospital production, the technology is characterized by means of a (minus) output distance function.^5^ Output distance functions should verify several properties [22]. In particular, a necessary condition to estimate output distance functions is linear homogeneity in outputs. This property could be imposed by choosing one output as the dependent variable and dividing the other outputs by this one.^6^ Therefore, the whole set of independent variables will include the inputs and the ratios of outputs defined to impose linear homogeneity in outputs. Hospital services, *y*_*l*_, are grouped into three outputs (*l* = 1,…, 3); and inputs, *x*_*j*_, (*j* = 1,…, 4) are aggregated into four categories, as described in the next section. Finally, a set of year dummies, *D*_*t*_, has been included to capture technical change in the hospitals over the period considered. Choosing a translog functional form, the model to be estimated is the following:

3 $$ \begin{gathered} \ln y_{1it} \; = \;\alpha_{0} \; + \;\sum\limits_{j\; = \;1}^{4} {a_{j} } \ln x_{jit} \; + \;\sum\limits_{l\; = \;2}^{3} {\beta {}_{l}\ln \left( {\frac{{y_{lit} }}{{y_{1it} }}} \right)} \; + \;\frac{1}{2}\;\sum\limits_{j\; = \;1}^{4} {\;\sum\limits_{k\; = \;1}^{4} {a_{jk} \;\ln \;x_{jit} \;\ln x_{kit} } } \; \\ + \;\frac{1}{2}\;\sum\limits_{l\; = \;1}^{3} {\;\sum\limits_{m\; = \;1}^{3} {\beta_{lm} \;\ln \;\left( {\frac{{y_{lit} }}{{y_{1it} }}} \right)\;\ln \left( {\frac{{y_{mit} }}{{y_{mit} }}} \right)} } + \;\frac{1}{2}\;\sum\limits_{j\; = \;1}^{4} {\;\sum\limits_{l\; = \;2}^{3} {\theta_{jl} \;\ln \;x_{jit} \;\ln \;\left( {\frac{{y_{lit} }}{{y_{1it} }}} \right)\;\sum\limits_{t\; = 2}^{T} {\alpha_{t} D_{t} } } } \; \\ + \;v_{it} - u_{it} \\ \end{gathered} $$

where *y*_*1it*_ is the output 1 (chosen as the dependent variable) of hospital *i* in year *t*; *x*_*jit*_ is the amount of input *j* used by hospital *i* in year *t; u*_*it*_ is a non-negative error term and *v*_*it*_ is a symmetric error term and *α’s; β’s* and $\theta$’s are the parameters to be estimated.^7^

From the definition of the translog output distance function, it is clear that if resident physicians were included in the equation as a standard input, then it would be implicitly assumed that there are feasible input endowments for which resident physicians are more productive than senior physicians, which is not compatible with our main hypothesis. This way, the marginal rate of substitution (MRS) between two different inputs (*x*_*j*_ and *x*_*k*_) would become:

4 $$ MRS_{jk} \; = \;\frac{{\left( {\beta_{i} \; + \;\sum\nolimits_{i\; = \;1}^{N} {\beta_{ij} } \ln x_{i} \; + \;\sum\nolimits_{k\; = \;2}^{M} {\beta_{il} } \ln \left( {\frac{{y_{l} }}{{y_{1} }}} \right)x_{i} } \right)}}{{\left( {\beta_{j} \; + \;\sum\nolimits_{h\; = \;1}^{N} {\beta_{ik} } \ln x_{i} \; + \;\sum\nolimits_{k\; = \;2}^{m} {\beta_{km} } \ln \left( {\frac{{y_{m} }}{{y_{1} }}} \right)x_{k} } \right)}}. $$

Hence, whatever the value of the technological parameters, the other input endowments and the output ratios, there would exist a set of endowments (*x*_*j*_, *x*_*k*_) for which *x*_*j*_ is more productive than *x*_*k*_, and another set of endowments for which *x*_*j*_ productivity is less than that of *x*_*k.*_. This would imply that there always exists some resident and senior physicians’ endowment such that resident physicians are more productive than seniors.^8^

Regarding Eq. (3), we assume that *v*_*it*_ $\approx iid N\left(0,{\sigma }_{v}^{2}\right)$ and *u*_*it*_ ${\approx iid N}^{+}\left(0,{\sigma }_{u}^{2}\right)$. Moreover, following [23], we allow for heteroskedasticity in the non-negative error term. We model the variance of *u* as a function of a set of covariates, *b*, that can influence the distance of hospitals to the frontier, with *δ* the set of parameters to be estimated. Increases in the variance of *u* represent increases in the distance to the frontier and vice versa [23].

5 $${u}_{h}\approx iid {N}^{+}\left(0,{\sigma }_{u}^{2}\right), {\sigma }_{u}=g\left(b,\delta \right).$$

In particular, the variables included to explain the variance in the *u* error term are hospital ownership (*Private* which takes value 1 if the hospital is privately owned and zero otherwise) and two variables related with the specialization of the hospital: the number of specialities treated in the hospital (*Specialities*) with a maximum value of four (general medicine, obstetrics, paediatrics and surgery) and a Herfindahl index measuring the concentration of hospital discharges in those specialities (*Herfindahl*). This index takes values between 0 and 1, ranging from a low concentration to a high concentration. This way, our hypothesis is that the larger the number of specialities and the lower the concentration in some of them, the larger is the collaboration among several specialities, which may improve the hospital technical efficiency. In particular, the impact of these variables on ${\sigma }_{u}^{2}$ is assumed to be:

6 $$ \ln \;\sigma_{{u_{it} }} \; = \;\delta_{0} \; + \;\delta_{P} \;Private_{i} \; + \;\delta_{P} \;Specialities_{it} \; + \;\delta_{H} \;Herfindahl_{it} . $$

Finally, it should be noted that there are two main differences between this model and the one proposed by [20]. First, in our model the differences in productivity between the two groups of workers (senior and resident physicians) are not constant as they depend on the values of a function of some hospital characteristics. Second, the model in this paper presents a two-component error term as it corresponds to its stochastic frontier structure.

## Data

We use Spanish data to carry out the empirical analysis. Data come from the “*Establecimientos Sanitarios con Régimen de Internado*” (In-Patient Health Care Establishments), compiled by the Spanish Ministry of Health and Consumption. This dataset includes the whole set of (private and public) general hospitals in Spain, with data spanning over the period 1997–2009.^9^

For the purpose of homogenizing the sample, we have excluded from the analysis those hospitals that have a specific medical vocation that cannot be considered to be that of a general hospital. Moreover, not all hospitals are observed during the sample period, with some of the disappearing and new ones being created. Moreover, during the period studied there have been merger activities among certain hospitals. In this case, the hospital resulting from the merger is considered a new hospital.

Only hospitals with intensive care units are considered. We have eliminated hospitals with less than 100 beds [24, 25]^10^ and those showing zero values for any of the relevant variables in our study. For each year, a hospital is considered to be a teaching hospital if it has resident physicians that year. As a result, the final sample is formed by an unbalanced panel that consists of 312 hospitals (corresponding to 3056 observations). Of these, 88 have been non-teaching hospitals in all years considered.

In Table 1 we display the summary statistics related to the number of residents in teaching hospitals.^11^ Medical residents in Spain hold special work contracts with teaching hospitals, which confer various rights and obligations. With respect to their rights, the contract has a maximum duration of 1 year and is renewed for similar time spans during the period covered by the residency programme, always provided that they pass the evaluations of the programme satisfactorily. The working day is designed in a manner which permits the doctor to fulfil the requirements of the training programmes. In addition to these rights, the resident is subject to a series of obligations. One of these is to accomplish the training programme on a full-time basis, without the option of exercising any other working activity, whether gainful or not, during the training period, even if this takes place outside the established working hours. With respect to the remuneration of doctors, this is the exclusive competence of the State, within a general framework and is the same for all personnel being completely independent of the centre responsible for their training.

**Table 1:** Number of resident physicians in teaching hospitals

Year	Teaching hospitals	Mean	Std. dev	Min	Max
1996	148	89.10	102.27	1	436
1997	150	87.14	95.50	1	436
1998	148	88.28	97.49	1	431
1999	149	87.44	98.40	1	415
2000	156	81.71	96.08	1	422
2001	152	76.41	95.82	1	413
2002	159	84.18	100.15	1	442
2003	160	84.48	101.46	1	469
2004	162	88.49	104.02	1	449
2005	156	94.01	106.08	2	471
2006	161	96.75	109.47	1	494
2007	162	99.06	109.13	1	469
2008	163	103.74	111.26	1	478
2009	166	105.78	113.42	1	477

Number of observations: 2192

**Fig. 1:**
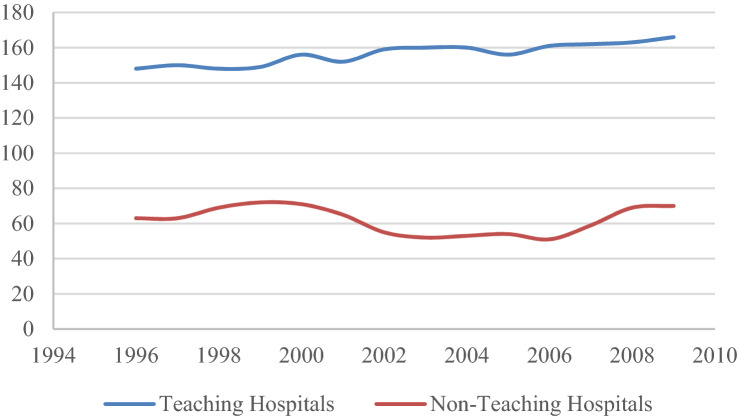
Evolution in the number of teaching and non-teaching hospitals

According to Table 1, and considering only teaching hospitals, the average number of resident physicians is 90.5 at the sample mean. In general terms, we note a slight decrease between 1999 and 2001, with this figure increasing towards the end of the sample period. Figure 1 shows the evolution of the number of teaching and non-teaching hospitals included in the sample during the period considered.

The summary statistics are displayed in Table 2. With respect to output measures, we aggregate hospital production into three output categories: non-intensive care discharges (*y*_*1*_), which is a weighted (by WCU) sum of discharges in general medicine, surgery, paediatric and obstetrics medicine; outpatient visits (*y*_*2*_), which is a weighted (by WCU) sum of first and successive visits and emergencies without entrance; and intensive-care discharges (*y*_*3*_).

**Table 2:** Summary statistics

Variable	Mean	Std. dev	Min	Max
${y}_{1}$	21,736	16,046	344	113,862
${y}_{2}$	185,901	162,617	209	993,690
${y}_{3}$	987	1160	1	11,803
Resident physicians	65	97	0	494
DOCT (*x*_1_)	257	207	11	1533
TECH (*x*_2_)	451	433	2	2424
BED (*x*_3_)	421	331	100	1789
SUPP (*x*_4_)	28	31	0.02	231
Efficiency determinants				
Private	0.254	0.435	0	1
Specialities	3.721	0.632	1	4
Herfindahl	0.381	0.128	0.254	1
Resident productivity determinants				
%ICU	0.042	0.028	0	0.287

Number of observations: 3056

With respect to the inputs, we consider four categories: doctors (DOC), which includes both senior and resident physicians as defined in Equations (1) and (2); care technicians (TECH), which includes nurses, matrons and others; endowment of beds (BED); and expenses on sanitary material, food, clothing, fuels and others (SUPP), measured in constant year 2006 euros (millions of euros).

**Fig. 2:**
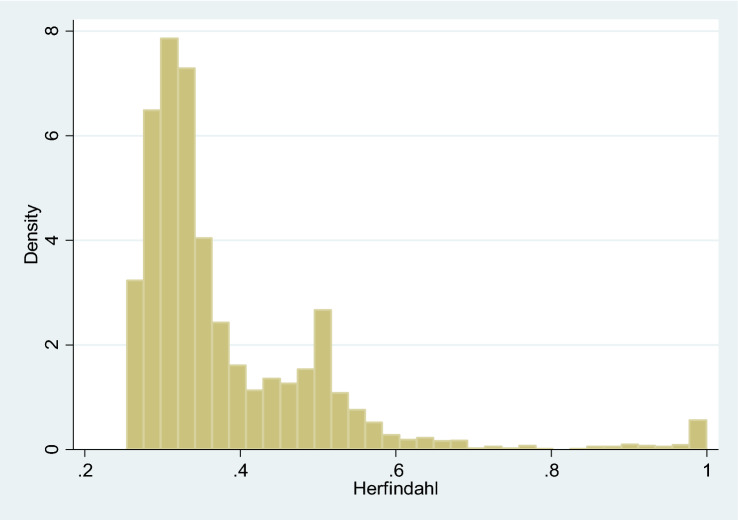
Histogram of Herfindahl index (TE)

Regarding the determinants of technical efficiency, approximately 75% of the hospitals in the sample are publicly owned. On the other hand, on average, hospitals operate in more than three specialities. Finally, the Herfindahl index in the sample mean is 0.38, which indicates, again, a low concentration index in our sample. Figure 2 shows the histogram for the values of the Herfindahl index. We can observe that for most of the observations, this index takes low values, indicating a low degree of concentration in our sample.

## Results

The model defined in Equation (3) is estimated by maximum likelihood using a stochastic frontier approach. Due to its non-linear nature in parameters, the stochastic frontier cannot be estimated using traditional statistical packages for these types of models, so it has been programmed using the GAUSS statistical package. Table 3 displays the results of estimating the (minus) output-oriented distance function. The equations are estimated centring the data at the sample geometric mean. Therefore, the first-order parameters estimated can be interpreted as the value of the corresponding elasticities evaluated at this point. Monotonicity conditions imply non-negative input marginal productivities and non-positive output elasticities.^12^ Hence, these restrictions are verified at the sample mean if the elasticities of inputs and outputs are positive and negative, respectively. Negative values for the elasticities of the outputs indicate that if the output increases, the “radial” distance to the production possibilities frontier decreases (that is, efficiency increases). From Table 3, it is clear that the elasticities have the expected sign and are highly significant. Thus, the estimated technology complies with the theoretically expected monotonicity restrictions at the sample mean.

**Table 3:** Multiple-output technology estimation

Variable	Value	Std. error	*t*-stat	Variable	Value	Std. error	*t*-stat
**Constant**	9.956	0.013	741.02	**ln *x***_**1**_**ln *x***_**2**_	0.035	0.015	2.27
**ln** ***y***_**2**_	− 0.242	0.008	− 31.16	**ln *x***_**1**_**ln *x***_**3**_	− 0.052	0.029	− 1.81
**ln** ***y***_**3**_	-0.021	0.007	− 2.95	**ln *x***_**1**_**ln *x***_**4**_	− 0.036	0.012	− 3.04
**ln** ***x***_**1**_	0.256	0.015	17.68	**ln *x***_**2**_**ln *x***_**3**_	0.124	0.026	4.72
**ln** ***x***_**2**_	0.063	0.015	4.25	**ln *x***_**2**_**ln *x***_**4**_	− 0.287	0.021	− 13.66
**ln** ***x***_**3**_	0.362	0.014	25.25	**ln *x***_**3**_**ln *x***_**4**_	0.238	0.026	9.33
**ln** ***x***_**4**_	0.176	0.008	21.11	**D**_**1997**_	− 0.001	0.016	− 0.04
**ln** ***y***_**2**_^**2**^	− 0.056	0.005	− 10.81	**D**_**1998**_	0.014	0.017	0.85
**ln** ***y***_**3**_^**2**^	− 0.031	0.006	-4.96	**D**_**1999**_	0.014	0.017	0.79
**ln** ***x***_**1**_^**2**^	0.217	0.021	10.14	**D**_**2000**_	0.021	0.017	1.24
**ln** ***x***_**2**_^**2**^	0.149	0.018	8.11	**D**_**2001**_	0.029	0.016	1.83
**ln** ***x***_**3**_^**2**^	− 0.445	0.044	− 10.12	**D**_**2002**_	0.025	0.017	1.49
**ln** ***x***_**4**_^**2**^	0.055	0.004	12.87	**D**_**2003**_	− 0.003	0.017	− 0.19
**ln** ***y***_**2**_**ln** ***y***_**3**_	0.085	0.006	14.99	**D**_**2004**_	− 0.018	0.017	− 1.05
**ln** ***y***_**2**_**ln** ***x***_**1**_	0.025	0.011	2.20	**D**_**2005**_	− 0.028	0.017	− 1.61
**ln** ***y***_**2**_**ln** ***x***_**2**_	− 0.123	0.015	− 8.22	**D**_**2006**_	− 0.049	0.019	− 2.66
**ln** ***y***_**2**_**ln** ***x***_**3**_	0.005	0.014	0.36	**D**_**2007**_	− 0.071	0.017	− 4.08
**ln** ***y***_**2**_**ln** ***x***_**4**_	0.129	0.012	10.45	**D**_**2008**_	− 0.076	0.017	− 4.41
**ln** ***y***_**3**_**ln** ***x***_**1**_	0.020	0.010	1.92	**D**_**2009**_	− 0.080	0.017	− 4.60
**ln** ***y***_**3**_**ln** ***x***_**2**_	− 0.030	0.010	− 3.00	**ln σ**_**v**_	− 2.220	0.030	− 73.69
**ln** ***y***_**3**_**ln** ***x***_**3**_	− 0.021	0.012	− 1.67	***γ***	0.693	0.137	5.07
**ln** ***y***_**3**_**ln** ***x***_**4**_	0.022	0.009	2.61	***γ***_**%ICU**_	− 7.623	1.613	− 4.73
**Log likelihood**	1088						

**Fig. 3:**
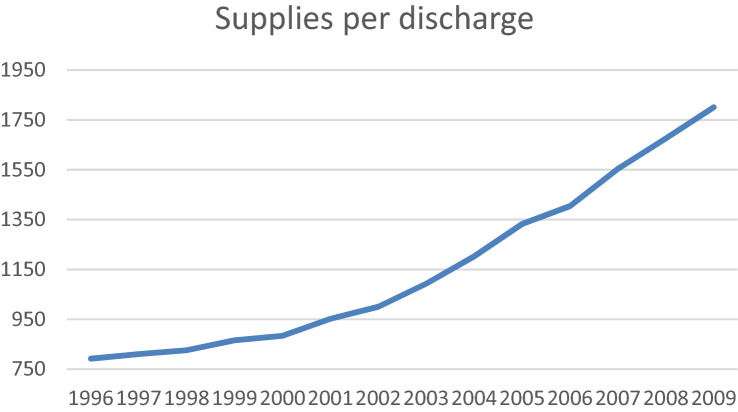
Evolution of supplies per discharge

According to the estimated coefficients of the year dummies, there seems to be evidence that during the sample period, more inputs are required to produce the output. While this result may appear surprising, a more thorough analysis of the data reveals that this can be explained by the use of more expensive (even in constant euro) medicine and equipment over time (which are measured in monetary terms). In this sense, Fig. 3 shows the evolution of supplies per discharges in our sample. The rapid pace of scientific advances is enabling the development of new medicines and this development is more intense as we go through time. However, the average cost to research and develop each new drug is very high and this may be causing the costs of hospital supplies to grow exponentially. Figure 3 shows this trend in our sample and may explain the significance of the temporal dummies at the end of the period.

Returns to scale are calculated as the sum of input elasticities [16]. At the sample mean, our model produces an average estimate of the returns to scale at about 0.86 and is significantly different from 1.^13^ Therefore, the estimated technology shows decreasing returns to scale.

Related with our main objective, the productivity of resident physicians relative to that of attending physicians *γ(z)* depends on the estimation of the parameters in Eq. (3), specifically on *γ* and *γ*_*%ICU*_. Both parameters are significant. First, the estimated value of *γ* is positive and lower than one, as expected. Second, *γ*_*%ICU*_ is negative and highly significant indicating that when the hospital treats highly complex cases (measured as the percentage of ICU with respect to the total of treated cases), medical students will reduce their productivity relative to that of senior doctors. From these results, we can calculate the value of *γ(z)* according to Eq. (2). The value of this expression is 0.37 at the sample mean, and significantly lower than one, as shown in Table 4. Therefore, resident physicians’ productivity is in average positive, but lower than that of senior physicians. Specifically, this result indicates that, on average, the productivity of a resident physician is 37% of that of a senior physician. Still, residents contribute positively to hospital production and, consequently, their net effect is that of an input.

**Table 4:** Heteroskedasticity of the *u* error term

Variable	Value	Std. error	*t*-stat
Constant	− 1.845	0.212	− 8.71
Private	0.069	0.038	1.81
Specialities	− 0.182	0.038	− 4.82
Herfindahl	2.488	0.219	11.35

**Fig. 4:**
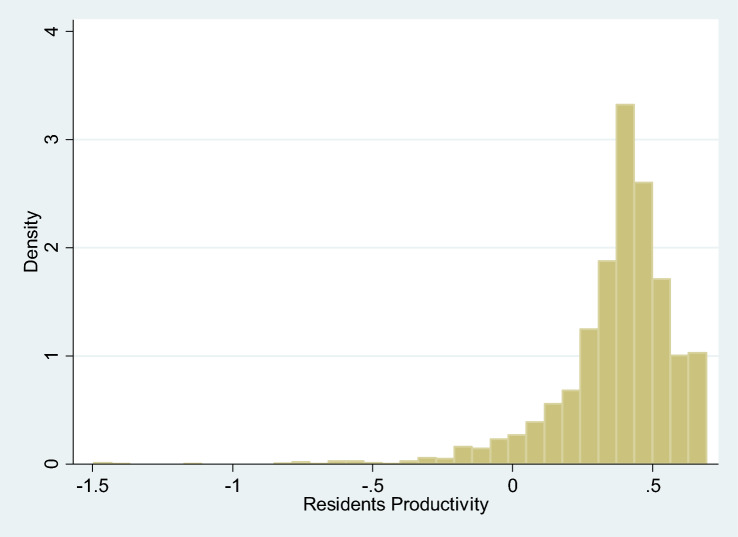
Histogram of residents’ productivity index ($\gamma \left(z\right)$)

Figure 4 shows the histogram for resident physicians’ productivity. For most of the observations, residents’ productivity, *γ(z)*, takes positive values (only in 5.73% of the cases is *γ(z)* negative, those in which the proportion of ICU is very high reaching values of over 9% of total discharges). Thus, these results confirm the negative relationship between *% ICU* and *γ(z)* already revealed by the coefficient *γ*_*%ICU*._

**Fig. 5:**
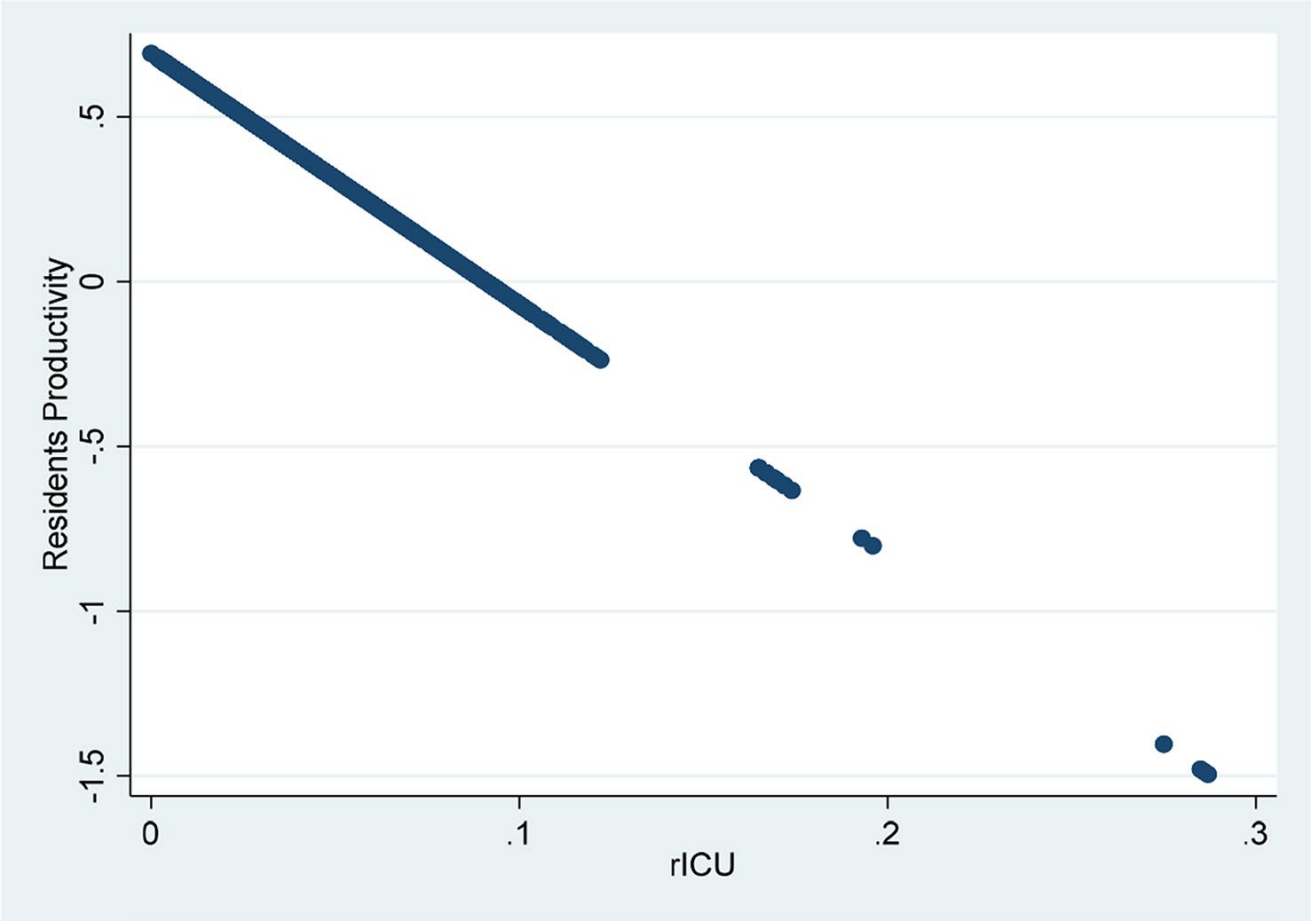
Relationship between residents’ productivity index ($\gamma \left(z\right)$) and rUCI

Moreover, as shown in Fig. 5, the lowest levels of productivity are observed for hospitals with a high degree of ICU activity, which corroborates our hypothesis of a relatively lower productivity of resident physicians when the case mix becomes more and more complex. Concretely, we have found that 5.7% of total observations have a productivity of resident physicians lower than zero (where only 16 observations corresponding to two hospitals are below − 0.24).

On the other hand, Table 5 exhibits the effect of the determinants of the heteroskedasticity of the *u* error term (Eq. 6). Recall that this term captures the difference between potential and observed productivity. A positive sign leads to an increase in the distance to the frontier (the hospital is less efficient). We find that the variance of the *u* error term is larger for privately owned hospitals, therefore presenting lower expected efficiency. This is a standard result in the literature (see for example, [26] for a review) that may be explained by factors, such as the care amenities offered by private hospitals or the fact that, usually, public hospitals face greater financial pressure so they tend to use their resources more efficiently. Conversely, hospitals with more medical specialities tend to be systematically closer to the frontier (more efficiency). In addition, the introduction of the Herfindahl index into the model indicates that the higher the hospital’s specialization, the lower is the technical efficiency index. These last two coefficients (specialities and Herfindahl) suggest that hospitals that diversify their activity can best take advantage of the synergies among the different medical specialities performed by the hospital. Therefore, a greater number of specialities can help improve efficiency, since it would imply a better use of resources. That is to say, collaboration among several specialists in the diagnosis/treatment of a patient can both save time and avoid duplication of diagnosis/treatment, thus improving the efficiency of the hospital (for example, doctors from two specialities can examine the same CAT—computed axial tomography—avoiding having to order a CAT for each department). These results confirm those found in other studies that find that less specialized hospitals are more efficient than the more specialized ones [19, 27].

**Table 5:** Descriptive statistics: resident’s productivity index

	Mean	Std. dev	Min	Max
$\gamma \left(z\right)$	0.373	0.194	− 1.495	0.693

**Fig. 6:**
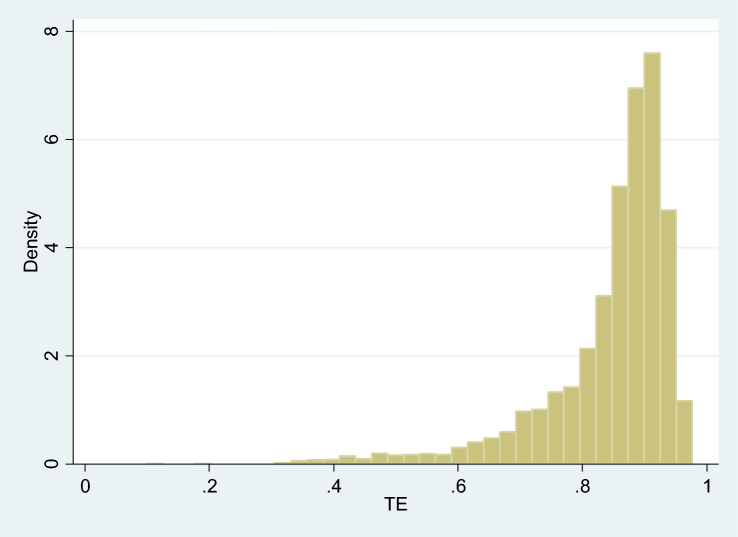
Histogram of technical efficiency index (TE)

From model (3), it is possible to calculate the technical efficiency indexes (TE) following [28]. Figure 6 and Table 6 show the histogram and descriptive statistics for technical efficiency indices, respectively. At the sample mean, technical efficiency is around 0.85% indicating that hospitals (on the data average) could increase their output by 15% with the available resources and technology.

**Table 6:** Descriptive statistics: technical efficiency index

	Mean	Std. dev	Min	Max
$\mathrm{TE}$	0.846	0.106	0.100	0.977

Finally, the Appendix shows the results of estimating the output distance function without including the resident physicians. It is worth noting that there are no significant differences in the elasticities between the two estimated models, which confirms the robustness of the estimated non-lineal model proposed in this study. The likelihood ratio test comparing both estimations reaches a value of 34.5, which is significant at any standard level showing, therefore, that the model including resident physicians is statistically preferred. In conclusion, resident physicians’ productivity is positive in most of the observations in our sample and its relative performance referred to the senior physicians’ one depends in the complexity of the hospital case mix.

## Conclusions

While in the literature there is no consensus as to the specific role of residents in the hospital production process, we argue that they should be considered as both inputs and outputs. Accordingly, we develop a model that allows us to distinguish whether the net effect of resident physicians on hospital production is that of an input or an output. Differences in productivity between senior and resident physicians are allowed to depend on the complexity of the case mix treated in the hospital. Additionally, if the parameters estimation verify some conditions the model proposed rules out the possibility that residents could be more productive than senior physicians for any set of medical inputs endowments.

Our results reveal that the contribution of resident physicians to the production of health services is larger than their consumption of inputs as educational output as the net effect of residents on hospital productivity is positive in most cases and, therefore, they should be overall considered as an input.

It is worth noting also that results corroborate our hypothesis that, on the average, residents are less productive than senior physicians and their relative productivity tends to diminish when the complexity of the case mixed treated in the hospital increases. In particular, we estimate that residents’ productivity is, on average, 37% of that of a senior physician. However, more research is needed to improve knowledge on this matter. In this regard, the availability of data about the resident physicians' years of residence as well as their speciality would make possible a better approximation of their marginal productivity. This would help improve hospital management to the extent that a more accurate estimation of their real contribution to hospital activity could be obtained.

## Appendix

Tables 7, 8.

**Table 7:** Distance function estimation without resident physicians

Variable	Value	Std. error	*t*-stat	Variable	Value	Std. error	*t*-stat
**Constant**	9.961	0.014	732.44	**ln ** ***x***_**1**_^*****^ **ln ** ***x***_**2**_	0.047	0.015	3.09
**ln** ***y***_**2**_	− 0.246	0.008	− 31.71	**ln ** ***x***_**1**_^*****^ **ln ** ***x***_**3**_	− 0.091	0.027	− 3.40
**ln** ***y***_**3**_	− 0.038	0.005	− 7.13	**ln ** ***x***_**1**_^*****^ **ln ** ***x***_**4**_	− 0.036	0.012	− 2.98
**ln** ***x***_**1**_^*****^	0.232	0.014	16.71	**ln ** ***x***_**2**_ **ln ** ***x***_**3**_	0.137	0.026	5.23
**ln** ***x***_**2**_	0.078	0.015	5.14	**ln ** ***x***_**2**_ **ln ** ***x***_**4**_	− 0.295	0.021	− 14.32
**ln** ***x***_**3**_	0.367	0.014	27.10	**ln ** ***x***_**3**_ **ln ** ***x***_**4**_	0.254	0.024	10.43
**ln** ***x***_**4**_	0.188	0.008	23.39	**D**_**1997**_	0.000	0.017	− 0.01
**ln** ***y***_**2**_^**2**^	− 0.057	0.005	− 11.00	**D**_**1998**_	0.013	0.017	0.79
**ln** ***y***_**3**_^**2**^	− 0.037	0.005	− 7.15	**D**_**1999**_	0.013	0.018	0.72
**ln** ***x***_**1**_^***2**^	0.222	0.022	10.07	**D**_**2000**_	0.018	0.017	1.06
**ln** ***x***_**2**_^**2**^	0.147	0.019	7.96	**D**_**2001**_	0.024	0.016	1.47
**ln** ***x***_**3**_^**2**^	− 0.432	0.043	− 9.99	**D**_**2002**_	0.022	0.017	1.30
**ln** ***x***_**4**_^**2**^	0.056	0.004	13.21	**D**_**2003**_	− 0.008	0.017	− 0.49
**ln** ***y***_**2**_**ln** ***y***_**3**_	0.088	0.006	15.68	**D**_**2004**_	− 0.024	0.017	− 1.38
**ln** ***y***_**2**_**ln** ***x***_**1**_^*****^	0.021	0.011	1.83	**D**_**2005**_	− 0.035	0.018	− 1.99
**ln** ***y***_**2**_**ln** ***x***_**2**_	− 0.118	0.015	− 7.88	**D**_**2006**_	− 0.056	0.019	− 3.01
**ln** ***y***_**2**_**ln** ***x***_**3**_	0.002	0.014	0.12	**D**_**2007**_	− 0.079	0.018	− 4.49
**ln** ***y***_**2**_**ln** ***x***_**4**_	0.128	0.012	10.47	**D**_**2008**_	− 0.084	0.017	− 4.85
**ln** ***y***_**3**_**ln** ***x***_**1**_^*****^	0.017	0.010	1.62	**D**_**2009**_	− 0.089	0.018	− 5.07
**ln** ***y***_**3**_**ln** ***x***_**2**_	− 0.032	0.010	− 3.30	**ln σ**_**v**_	− 2.208	0.029	− 75.41
**ln** ***y***_**3**_**ln** ***x***_**3**_	− 0.031	0.012	− 2.69	***γ***	–	–	–
**ln** ***y***_**3**_**ln** ***x***_**4**_	0.020	0.008	2.36	***γ***_**%ICU**_	–	–	–
**Log likelihood**	1071						

**Table 8:** *u* Heteroskedasticity without resident physicians

Variable	Value	Std. error	*t*-stat
Constant	− 1.837	0.211	− 8.71
Private	0.077	0.039	2.00
Specialities	− 0.179	0.038	− 4.77
Herfindahl	2.444	0.218	11.21

## Footnotes

1 That is, we assume that resident physicians are not as productive as they become when finalizing their training process. This implies that, on average, resident physicians are not so productive as senior physicians.

2 We thank an anonymous referee for suggesting this alternative interpretation of the medical labour input.

3 From the pioneering work of [29], the WCU has been used both in different academic studies [30, 31] and by the Spanish Ministry of Health, which uses it to calculate economic indicators such as budget or expenditure per activity (where hospital activity is weighted by WCU). The WCU takes into account the consumption of resources of each hospital activity, and with this information, weighs the different activities taking as reference a medical stay (that is to say, a medical stay = 1 WCU). From here, any activity becomes a UPA as follows: UCI (5.8 WCU); Surgery (1.5 WCU); Paediatric medicine (1.3 WCU); Obstetrics medicine (1.2 WCU); first outpatient visit (0.25); second and successive outpatient visits (0.15 WCU) and emergencies without entrance (0.3 WCU)”.

4 That is to say*: **r *$ICU=\frac{ICU discharges}{Discharges}$; where: ${\text{Discharges }} = {\text{ General Medicine}} + 1.5{\text{ Surgery}} + 1.3{\text{ Paediatric Medicine}} + 1.2{\text{ Obstetric Medicine}}$

5 The dependent variable in the output distance function would be $-\mathrm{ln}{y}_{1}$. Using $\mathrm{ln}{y}_{1}$ as the dependent variable, the sign of the estimated parameters changes allowing a more standard interpretation.

6 Results are invariant to the output chosen to impose homogeneity.

7 Note that *x*_*1it*_ is not a standard input as it is defined through Eqs. (1) and (2) which produces a highly non-linear (in parameters) specification of the frontier technology.

8 This is especially clear if the second order terms in the translog function are null and then, technology becomes a Cobb–Douglas one.

9 From 2009 on the survey was modified. With the new survey, essential information for an unbiased approach to technology (such as the separation between general and non-general hospitals) is not available.

10 [24, 25] point out that there is evidence that hospitals with less than 100 beds exhibit cost structures that are different from larger hospitals.

11 Unfortunately, the data offered by the Spanish Ministry of Health only reports the number of resident physicians in each hospital, without specifying year and specialty. Because of this, it has not been possible to analyse the characteristics of these residents in more detail.

12 The use of a proper output distance function would require the opposite restrictions. Therefore, the use of the minus output distance function allows a more straightforward interpretation similar to that in a single output case.

13 The Wald test shows a value of 409 which is significant at any standard level. As [32] point out, studies in the literature have produced mixed and largely inconclusive results to the presence of hospital scale economies, with most of them finding limited evidence of scale economies beyond around 200 beds.

## References

[1] Grosskopf S, Margaritis D, Valdmanis V (2001). The effects of teaching on hospital productivity. Socioecon. Plann. Sci..

[2] Ferrier GD, Leleu H, Valdmanis VG, Vardanyan M (2018). A directional distance function approach for identifying the input/output status of medical residents. Appl. Econ..

[3] Custer WS, Willke RJ (1991). Teaching hospital costs: the effects of medical staff characteristics. Health Serv. Res..

[4] López-Casasnovas G, Saez M (1999). The impact of teaching status on average costs in Spanish hospitals. Health Econ..

[5] Rodríguez-Álvarez A, Knox Lovell CA (2004). Excess capacity and expense preference behaviour in national health systems: an application to the Spanish public hospitals. Health Econ..

[6] Jensen GA, Morrisey MA (1986). The role of physicians in hospital production. Rev. Econ. Stat..

[7] Lehner LA, Burgess JF (1995). Teaching and hospital production: the use of regression estimates. Health Econ..

[8] Campbell CR, Gillespie KN, Romeis JC (1991). The effects of residency training programs on the financial performance of veterans affairs medical centers. Inquiry.

[9] Sloan FA, Feldman RD, Steinwald AB (1983). Effects of teaching on hospital costs. J. Health Econ..

[10] Simmer TL, Nerenz DR, Rutt WM, Newcomb CS, Benfer DW (1991). A randomized, controlled trial of an attending staff service in general internal medicine. Med. Care..

[11] Cameron JM (1985). The indirect costs of graduate medical education. N. Engl. J. Med..

[12] Bhat R, Dubin J, Maloy K (2014). Impact of learners on emergency medicine attending physician productivity. West. J. Emerg. Med..

[13] Clinkscales JD, Fesmire FM, Hennings JR, Severance HW, Seaberg DC, Patil N (2016). The effect of emergency medicine residents on clinical efficiency and staffing requirements. Acad. Emerg. Med..

[14] Layer K, Johnson AL, Sickles RC, Ferrier GD (2019). Direction selection in stochastic directional distance functions. Eur. J. Oper. Res..

[15] Grosskopf S, Margaritis D, Valdmanis V (2001). Comparing teaching and non-teaching hospitals: a frontier approach (teaching vs. non-teaching hospitals). Health Care Manag. Sci..

[16] Färe R, Primont D (1995). Multi-output production and duality: theory and applications.

[17] Xenos P, Yfantopoulos J, Nektarios M, Polyzos N, Tinios P, Constantopoulos A (2017). Efficiency and productivity assessment of public hospitals in Greece during the crisis period 2009–2012. Cost Eff. Res. Alloc..

[18] Yildiz MS, Heboyan V, Khan MM (2018). Estimating technical efficiency of Turkish hospitals: implications for hospital reform initiatives. BMC Health Serv. Res..

[19] Colombi R, Martini G, Vittadini G (2017). Determinants of transient and persistent hospital efficiency: the case of Italy. Health Econ. (U. K.)..

[20] Hellerstein JK, Neumark D, Troske KR (1999). Wages, productivity, and worker characteristics: evidence from plant-level production functions and wage equations. J. Law Econ..

[21] Solow RM (1957). Technical change and the aggregate production function. Rev. Econ. Stat..

[22] Kumbhakar S, Lovell CAK (2000). Stochastic frontier analysis.

[23] Caudill SB, Ford JM, Gropper DM (1995). Frontier estimation and firm-specific inefficiency measures in the presence of heteroscedasticity. J. Bus. Econ Stat..

[24] Carey K (1997). A panel data design for estimation of hospital cost functions. Rev. Econ. Stat..

[25] Burgess JF, Wilson PW (1998). Variation in inefficiency among US hospitals. Infor.

[26] Asbu EZ, Masri MD, Naboulsi MA (2020). Determinants of hospital efficiency: a literature review. Int. J. Healthcare..

[27] Carey K, Burgess JF, Young GJ (2008). Specialty and full-service hospitals: a comparative cost analysis. Health Serv. Res..

[28] Jondrow J, Lovell CAK, Materov IS, Schmidt P (1982). On the estimation of technical inefficiency in the stochastic frontier production function model. J. Econometrics..

[29] Bestard Perelló J, Sevilla Pérez F, Corella Monzón I, Elola Somoza J (1993). La unidad ponderada asistencial (UPA): nueva herramienta para, la presupuestación hospitalaria. Gac. Sanit..

[30] Feldman R, Lobo F (1997). Global budgets and excess demand for hospital care. Health Econ..

[31] García-Gómez M, Urbanos Garrido R, Castañeda López R, Menéndez-Navarro A (2017). Medical costs of asbestos-related diseases in Spain between 2004 and 2011. Ind. Health.

[32] Gaynor MS, Kleiner SA, Vogt WB (2015). Analysis of hospital production: an output index approach. J. Appl. Economet..

